# Rapid progression of non-culprit vessels (NCVs) post-PCI in acute myocardial infarction: Causes and current research status

**DOI:** 10.1097/MD.0000000000046763

**Published:** 2026-01-09

**Authors:** Xingpo Li, Zishan Wang, Shanshan Wang, Xue Xu, Bin Yi, Hongxia Yu

**Affiliations:** a Department of Cardiology, Huantai County Hospital of Traditional Chinese Medicine, Zibo, Shandong, China; b Department of Cardiology, Zibo Hospital of Integrated Traditional Chinese and Western Medicine, Zibo, Shandong, China.

**Keywords:** acute myocardial infarction, non-culprit vessels, progression

## Abstract

Acute myocardial infarction (AMI) remains a major cause of morbidity and mortality globally, with percutaneous coronary intervention (PCI) serving as the primary treatment for revascularization of culprit vessels. However, non-culprit vessels (NCVs), which harbor significant atherosclerosis but are not directly responsible for acute ischemic events, have gained increasing attention owing to their role in future cardiovascular risks. Recent studies have highlighted the rapid progression of post-PCI, driven by factors such as inflammation, endothelial dysfunction, and hemodynamic changes. This progression is linked to adverse outcomes, including recurrent myocardial infarction and cardiovascular mortality. Inflammation, particularly activation of the NLRP3 inflammasome, plays a critical role in plaque vulnerability within NCVs, and is exacerbated by systemic responses following PCI. Endothelial dysfunction, characterized by reduced nitric oxide availability and oxidative stress, further accelerates atherosclerotic progression in NCVs. Imaging technologies such as optical coherence tomography and intravascular ultrasound allow for a detailed assessment of these vessels, enabling the identification of high-risk plaques. Pharmacological interventions, such as high-intensity statins, PCSK9 inhibitors, and anti-inflammatory therapies targeting IL-1β, are emerging as promising strategies to mitigate NCV progression. Complete revascularization, addressing both culprit and NCVs, may improve the long-term outcomes in patients with AMI. Future research should focus on understanding the pathophysiology of NCVs, optimizing therapeutic strategies, and personalizing treatment approaches based on patient risk profiles to enhance the management and prognosis of patients with AMI.

## 1. Introduction

### 1.1. Background of acute myocardial infarction (AMI)

Acute myocardial infarction (AMI) remains a leading cause of morbidity and mortality worldwide, placing a significant burden on healthcare systems and affecting patients’ quality of life.^[1,2]^ Epidemiological studies have indicated an increasing incidence of AMI, particularly among older adults and individuals with risk factors such as diabetes, hypertension, and hyperlipidemia. In developed nations, the prevalence of AMI ranges from 0.5% to 1.5% annually,^[3]^ with developing countries experiencing increasing trends due to urbanization and lifestyle changes. A thorough understanding of AMI epidemiology is crucial for implementation of effective prevention and management strategies.

In the context of coronary intervention for AMI, the terms “culprit” and “non-culprit” vessels are commonly used. The culprit vessel refers to the artery responsible for an acute ischemic event that is typically identified during coronary angiography.^[4]^ In contrast, non-culprit vessels (NCVs) may harbor significant atherosclerotic plaques but are not directly responsible for myocardial infarction. Revascularization of the culprit lesion via percutaneous coronary intervention (PCI) is the cornerstone of AMI management and is linked to improved short- and long-term outcomes.^[5]^ While restoring blood flow in the culprit vessel is crucial, the condition of NCVs also significantly affect the overall prognosis. Rapid disease progression in these vessels can lead to recurrent myocardial infarction, underscoring the need for vigilant monitoring and management.

Current research on the NCVs in AMI is evolving. Although many studies have focused on immediate outcomes after post-PCI of the culprit vessel, there is growing recognition of the importance of NCVs in the long-term prognosis of patients with AMI. Recent findings suggest that NCVs can rapidly progress following an acute event, influenced by factors such as inflammation, hemodynamic changes, and systemic responses after post-PCI.^[6,7]^ Significant NCVs were associated with worse clinical outcomes, including higher rates of recurrent adverse cardiovascular events. Despite emerging evidence, consensus on optimal management strategies for NCVs remains elusive, necessitating further research to understand their pathophysiology and mitigate the associated risks.

AMI presents substantial challenges for both immediate and long-term management. A comprehensive understanding of AMI epidemiology, the roles of culprit and NCVs, and current research on NCV progression are essential for clinicians. This knowledge will aid in refining the treatment strategies to improve patient outcomes and prevent future cardiovascular events.

PCI has transformed the management of AMI and has revolutionized cardiovascular care. PCI, a minimally invasive procedure, is pivotal for urgent revascularization of occluded coronary arteries, restoration blood flow to the myocardium, and minimization of ischemic damage. Timely PCI is crucial, as studies have shown that each minute of delay significantly increases myocardial necrosis and worsens clinical outcomes.^[8]^ Owing to its ability to quickly relieve symptoms and improve survival, PCI is the standard of care for patients with ST-elevation myocardial infarction and non-ST-elevation myocardial infarction. In AMI, the coronary artery vessels are categorized as either culprit or non-culprit. The culprit vessel is an artery that is acutely occluded by thrombus formation, causing myocardial infarction.^[8,9]^ Identifying and successfully treating these vessels are essential for effective care. In contrast, NCVs may have significant atherosclerotic plaques but do not cause immediate ischemic event. However, these vessels still pose a significant risk for future cardiovascular events, as their condition can worsen after the acute phase of AMI.^[10]^ The significance of PCI extend beyond treating the culprit lesion and involves important considerations for managing NCVs. Although the primary goal of PCI is to restore blood flow to the myocardium, clinicians must monitor NCVs, which may worsen post-PCI owing to inflammatory and hemodynamic changes. Understanding the dynamics between culprit and NCVs is essential for developing strategies to treat acute events and mitigate future risks of coronary artery disease.^[11]^ PCI is a cornerstone of AMI management that significantly improve patient outcomes through rapid revascularization of the culprit vessel.^[12]^ However, NCVs must be carefully considered, as they are crucial for long-term prognosis and patient care. As our understanding of these dynamics evolves, it will enable the development of better strategies to optimize AMI management and improve cardiovascular health.

### 1.2. Significance of studying non-culprit vessel progression

The management of NCVs in AMI patients has garnered attention recently, as research has revealed the accelerated progression of these vessels post-intervention. Recognizing the clinical importance of this rapid progression is critical for improving patient outcomes. NCVs, often containing significant atherosclerosis, may lead to adverse cardiovascular events, such as reinfarction, owing to their instability. Studies indicate that 20% to 30% of patients experience events related to NCVs within a year following the initial AMI,^[13–15]^ emphasizing the necessity for vigilant monitoring and management.

Several factors contribute to the deterioration of the NCVs after post-PCI. The inflammatory response induced by myocardial infarction, coupled with hemodynamic changes resulting from successful revascularization, creates a hostile environment for these vessels. Traditional risk factors, including hypertension, hyperlipidemia, and diabetes mellitus, further increase plaque vulnerability in NCVs.^[16]^ Identifying these factors forms the basis for developing improved management strategies to mitigate risks associated with NCVs.

Recent research has shifted towards interventions targeting NCVs, exploring the feasibility and outcomes of performing PCI on these vessels during the initial procedure, particularly in high-risk patients. Evidence suggests that proactive intervention in significant NCVs may reduce recurrent myocardial infarction rates and enhance overall survival. Advances in imaging techniques such as optical coherence tomography (OCT) and intravascular ultrasound (IVUS)^[17]^ enable better characterization of NCVs, facilitating more precise decision-making regarding the necessity and timing of interventions. The progression of NCVs is notably rapid within the first 6 weeks post-PCI, with a marked peak in the early months following the procedure.^[18]^

In conclusion, managing NCVs in AMI is an evolving field with significant implications for patient care. Recognizing the clinical importance of accelerated lesion progression and understanding the factors contributing to instability allow healthcare providers to implement more effective management strategies.^[19]^ As research further elucidates the best practices for NCV interventions, the prognosis for patients with AMI is expected to improve, leading to better cardiovascular health.

## 2. Pathophysiology of non-culprit vessel progression

### 2.1. Inflammatory mechanisms

The progression of NCVs after AMI is strongly influenced by inflammatory mechanisms. Following an acute ischemic event, an inflammatory cascade is triggered, affecting both the culprit and NCVs. The underlying processes involve a complex interaction between immune cells, cytokines, and biochemical pathways, leading to plaque vulnerability and destabilization. Damage-associated molecular patterns released from necrotic myocardium activate the immune system, recruiting inflammatory cells, including macrophages, neutrophils, and T lymphocytes, to both affected and non-culprit coronary arteries.^[20]^ Macrophages, while clearing debris from ruptured plaques, produce pro-inflammatory cytokines such as tumor necrosis factor-alpha (TNF-α) and interleukin-1 beta (IL-1β), promoting inflammation and atherosclerosis progression in NCVs.^[21–23]^

Recent research has focused on identifying the specific inflammatory pathways involved in the progression of NCVs, notably the NLRP3 inflammasome, a crucial component of the innate immune response.^[18]^ Activation of the NLRP3 inflammasome results in the production of IL-1β, perpetuating inflammation and influencing smooth muscle cell proliferation and migration, contributing to plaque formation in NCVs.^[24]^ Additionally, systemic inflammatory markers such as C-reactive protein (CRP) correlate with the extent of atherosclerosis in NCVs and serve as potential prognostic indicators.

Understanding these inflammatory mechanisms opens new avenues for therapeutic interventions. Targeting the inflammatory pathways may stabilize non-culprit plaques and lower the risk of future cardiovascular events. Anti-inflammatory therapies, including monoclonal antibodies targeting IL-1β or TNF-α, have shown promise in clinical trials for reducing cardiovascular risk in patients.^[25,26]^ Lifestyle changes that lower systemic inflammation, such as dietary adjustments, exercise, and smoking cessation, may offer additional benefits in managing patients with AMI.

### 2.2. Endothelial dysfunction

The progression of NCVs post-AMI is heavily influenced by endothelial dysfunction, which is characterized by an imbalance between vasodilators and vasoconstrictors, increased permeability, and a pro-inflammatory state. Endothelial cells maintain vascular homeostasis, and their dysfunction can exacerbate atherosclerosis, leading to complications in patients.^[27]^

Endothelial dysfunction in NCVs can result from systemic inflammatory responses post-AMI, which release pro-inflammatory cytokines and oxidative stress markers that directly damage endothelial cells. Elevated levels of reactive oxygen species cause oxidative damage, reducing the production of nitric oxide (NO), a vital vasodilator and anti-inflammatory agent. Reduced NO availability promotes a pro-thrombotic and pro-inflammatory state, supporting plaque progression in non-culprit arteries.^[28]^

Risk factors such as hypertension, diabetes, and hyperlipidemia contribute to endothelial dysfunction through various mechanisms. Chronic hyperglycemia in diabetes results in glycation end products that damage endothelial cells, while elevated lipid levels lead to lipid accumulation, triggering inflammation and promoting atherogenesis.^[29]^ Advanced imaging techniques such as IVUS and OCT allow clinicians to observe structural changes in the endothelium, revealing significant remodeling and early plaque formation in NCVs.

Understanding the role of endothelial dysfunction in NCV progression underscores the potential of therapies that restore endothelial function. Lifestyle modifications such as diet and exercise can improve endothelial function and reduce the risk of future cardiovascular events. Additionally, drugs such as statins and ACE inhibitors not only treat dyslipidemia and hypertension but also improve endothelial function, making them key components in post-AMI management.

### 2.3. Pathological mechanisms: the role of unstable plaques

Unstable plaques are key factors in the progression of NCVs in AMI. Characterized by a thin fibrous cap, large lipid core, and elevated inflammatory cell infiltration, these plaques are prone to rupture, leading to thrombus formation and cardiovascular events even in arteries not initially identified as culprits in myocardial infarction.^[30]^

Advanced imaging techniques like OCT and IVUS have provided insights into the morphological changes in these plaques, showing that many NCVs possess high-risk features, including positive remodeling, spotty calcification, and a large lipid core. Identifying these features is valuable for prognosis, enabling the stratification of patients based on their risk of future cardiovascular events.

Therapeutic strategies to stabilize these plaques are currently being investigated. Statins reduce the lipid content and inflammation within plaques, stabilize their structure and lower the risk of rupture. Anti-inflammatory therapies, such as monoclonal antibodies targeting specific cytokines, are being explored for their potential to reduce plaque instability.^[31]^ Additionally, lifestyle changes, including dietary modifications and increased physical activity, have been shown to improve plaque stability and reduce cardiovascular risks.

The progression of NCVs following PCI has garnered increasing attention because of its impact on long-term cardiovascular outcomes. Several studies have identified that NCVs, although not responsible for the ischemic events, are vulnerable to rapid atherosclerotic progression after post-PCI. Clinical research has shown that the most significant progression typically occurs within the first year after PCI, with a pronounced peak in the first few months post-intervention.

A retrospective study involving 311 patients with acute coronary syndrome demonstrated that NCVs can experience substantial progression within 6 weeks post-PCI.^[32]^ This finding highlights the critical early period during which NCVs are at a high risk of adverse events. Furthermore, this progression of NCVs is closely associated with an increased risk of recurrent myocardial infarction and the need for repeat revascularization. In particular, within 1 year post-PCI, the incidence of repeat PCI in patients with progressing NCVs reached 14%, with further increases observed in subsequent years.

Additionally, the presence of high levels of inflammatory markers such as elevated levels of CRP and interleukin-6 (IL-6), has been linked to the progression of atherosclerosis in NCVs. These systemic markers reflect an ongoing inflammatory response that exacerbates the endothelial dysfunction and plaque instability. Consequently, patients with higher inflammatory markers within the first year after PCI are more likely to experience accelerated disease progression in NCVs, emphasizing the need for vigilant monitoring during this critical time period.

### 2.4. Hemodynamic changes post-PCI

Post-PCI hemodynamic instability is characterized by fluctuations in blood pressure, heart rate, and cardiac output, which affect the vascular environment. Several factors contribute to these changes, including reperfusion injury, altered shear stress, and autonomic dysregulation. Reperfusion injury, associated with oxidative stress and endothelial dysfunction, exacerbates the inflammatory response, and contribute to plaque instability in NCVs.^[33]^ Altered shear stress, mechanical intervention during PCI may alter shear stress on the vessel walls. These changes can affect endothelial cell function and promote a pro-atherogenic environment, thereby facilitating the progression of existing plaques in NCVs. Autonomic dysregulation, PCI may induce alterations in autonomic regulation and baroreceptor function, leading to episodic hypertension and hypotension. Such fluctuations can compromise vascular integrity and promote plaque vulnerability. Several factors including patients procedural and pharmacological interventions, can influence the degree of hemodynamic instability observed post-PCI, include patient, procedural, pharmacological interventions.

Patient-specific factors: preexisting conditions such as hypertension, diabetes, and heart failure may worsen hemodynamic instability. Age and baseline vascular health are crucial for determining a patient’s response to PCI. Procedural factors: the complexity of PCI, including the number of stents placed, length of the treated segment, and use of adjunctive therapies such as thrombectomy can affect post-procedural hemodynamics. Longer and more complex procedures are often associated with greater hemodynamic disturbances. Pharmacological interventions: medications administered during and after PCI, such as antiplatelets, anticoagulants, and vasodilators – can influence hemodynamic stability.^[34]^ Careful consideration of their choice and dosage is necessary to minimize adverse hemodynamic effects.

Recent research using advanced imaging techniques and computational fluid dynamics models has studied the effects of altered shear stress on plaque progression. These studies demonstrated that regions of low shear stress are more prone to plaque formation and instability, providing insights into the spatial distribution of vulnerable plaques in NCVs. This progression is closely associated with systemic inflammatory markers, including CRP and IL-6, which exacerbate endothelial dysfunction and plaque instability in NCVs.^[35]^

Clinical trials are currently exploring the benefits of optimizing hemodynamic parameters during and after PCI. For example, the use of intra-aortic balloon pumps and other mechanical circulatory support devices is being evaluated for their potential to stabilize hemodynamics and reduce the risk of adverse events in high-risk patients. Additionally, pharmacological advancements are being explored, with novel agents aimed at mitigating reperfusion injury and enhancing endothelial function.

## 3. Role of biomarkers in the progression of non-culprit vessels post-PCI

Biomarkers, particularly inflammatory markers such as CRP and IL-6, play a significant role in the progression of NCVs post-PCI. Elevated levels of these markers indicate systemic inflammation, contributing to the destabilization of atherosclerotic plaques in NCVs. CRP is an acute phase protein that increases in response to inflammation. Elevated CRP levels after PCI indicate an ongoing inflammatory response which can worsen endothelial dysfunction and lead to plaque instability. Elevated CRP levels after PCI indicate an ongoing inflammatory response which can worsen endothelial dysfunction and lead to plaque instability. IL-6 is a pro-inflammatory cytokine that is central to the inflammatory cascade. High levels of IL-6 after post-PCI are indicative of increased inflammatory activity. IL-6 stimulates the production of other inflammatory mediators and promotes the differentiation of monocytes into macrophages.^[36]^ These macrophages infiltrate atherosclerotic plaques, and increase their vulnerability. High CRP and IL-6 levels impair endothelial function by reducing NO availability and increasing oxidative stress. This dysfunction fosters a pro-thrombotic and pro-inflammatory environment, making plaques more likely to rupture. CRP and IL-6 contribute to plaque inflammation. CRP activates the complement pathways and enhances macrophage infiltration. IL-6 promotes the secretion of matrix metalloproteinases (MMPs), which degrade the extracellular matrix and thin the fibrous cap, increasing the risk of rupture.^[6]^

Recent studies have provided insights into the role of biomarkers in plaque instability. Advanced biomarker profiling techniques help identify patients at higher risk of NCV progression. Targeted therapies aimed at reducing these inflammatory biomarkers, including monoclonal antibodies against IL-6, have shown promise for reducing inflammation and stabilizing plaque structures. Additionally, novel biomarkers such as TNF-α monocyte chemoattractant protein-1, and soluble CD40 ligand are under investigation for their roles in plaque instability, offering new targets for therapy and risk stratification.^[37]^

## 4. Clinical outcomes and prognosis of rapid progression in non-culprit vessels

The rapid progression of NCVs after post-AMI has significant implications for clinical outcomes and prognosis. NCV progression is associated with increased all-cause mortality, cardiovascular mortality, recurrent myocardial infarction, and the need for repeat revascularization. Both short- and long-term prognostic assessments highlight the importance of early identification and aggressive management of patients with rapid NCV progression.

Research continues to explore the mechanisms underlying NCV progression and their impact on clinical outcomes. Advanced imaging techniques, such as IVUS, OCT, and fractional flow reserve, are being studied to identify high-risk plaques in NCVs and predict clinical outcomes. Pharmacologic interventions, including anti-inflammatory drugs, statins, and novel lipid-lowering therapies, are being evaluated for their efficacy in reducing NCV progression and improving clinical outcomes.

## 5. Prevention and management strategies for rapid progression of non-culprit vessels in AMI

### 5.1. Pharmacological treatment advances

Statins remain the cornerstone of pharmacological treatment to prevent NCV progression, and high-intensity statin therapy has been shown reduce LDL cholesterol levels and stabilize atherosclerotic plaques. Proprotein convertase subtilisin/kexin type 9 (PCSK9) inhibitors, such as alirocumab and evolocumab, have emerged as potent agents for lowering LDL cholesterol, with the potential to further reduce cardiovascular events.^[38]^ Anti-inflammatory agents, including IL-1β inhibitors like canakinumab, have demonstrated promise in reducing cardiovascular events by targeting the inflammatory cascade. Recent data from the CANTOS trial showed a significant reduction in cardiovascular events with the use of canakinumab, with an absolute risk reduction of 1.9% in recurrent myocardial infarction.^[16]^

### 5.2. Interventional strategies

The strategy of performing PCI during the acute phase of AMI has evolved to include considerations of NCVs. Complete revascularization, involving treatment of both culprit and NCVs, has shown promise in reducing the risk of cardiovascular death or myocardial infarction. This approach addresses the underlying atherosclerotic burden more comprehensively and potentially prevent rapid progression of untreated NCVs. The MULTISTARS AMI trial demonstrated that complete revascularization reduced the risk of recurrent MI by 10% in patients with multivessel disease.^[37]^

## 6. Novel therapeutic targets

Beyond their lipid-lowering effects, PCSK9 inhibitors are being investigated for their role in plaque stabilization and potential anti-inflammatory properties. Statins (especially high-intensity statins) and PCSK9 inhibitors (such as alirocumab and evolocumab) have shown significant clinical benefits in reducing NCV progression and further lowering the risk of cardiovascular events.^[39]^ Inclisiran is a PCSK9 siRNA that has achieved good results in the real world. The success of the CANTOS trial has spurred interest in other anti-inflammatory agents targeting the IL-1β pathway. Research into other inflammatory mediators, such as IL-6, TNF-α, and monocyte chemoattractant protein-1, with potential therapeutic options for preventing the rapid progression of atherosclerosis in NCVs is ongoing.

## 7. Current challenges and future research directions

The study of NCV progression in AMI presents several challenges, including heterogeneity in patient populations, variability in imaging techniques, and short follow-up periods. Future research should focus on standardizing imaging protocols, conducting long-term prospective studies, and integrating multimodal data for a comprehensive risk assessment.^[40]^ Exploring novel therapeutic targets and combination therapies, along with personalized medical approaches, promises to improve the prevention and management of NCV progression.

## 8. Conclusion

The rapid progression of NCVs post-AMI presents a complex challenge in clinical practice, with implications for patient outcomes. This multifactorial phenomenon arises from a combination of factors that significantly contribute to the atherosclerotic process, including inflammation and endothelial dysfunction, future research should aim to elucidate the mechanisms underlying NCV progression, focusing on inflammatory pathways, endothelial health, and other contributing factors. Personalized treatment strategies that integrate clinical, imaging, and biomarker data, are essential for identifying high-risk patients and implementing targeted interventions.^[41]^ By advancing our understanding of NCV progression, we can improve the management of AMI patients and reduce the incidence of recurrent cardiovascular events.

## Author contributions

**Conceptualization:** Xingpo Li.

**Data curation:** Xingpo Li.

**Formal analysis:** Xingpo Li, Xue Xu.

**Project administration:** Hongxia Yu.

**Resources:** Zishan Wang.

**Software:** Xue Xu.

**Supervision:** Bin Yi.

**Validation:** Zishan Wang.

**Visualization:** Shanshan Wang.

**Writing – original draft:** Hongxia Yu, Bin Yi.

**Writing – review & editing:** Hongxia Yu.
